# TRPA1 as a O_2_ sensor detects microenvironmental hypoxia in the mice anterior cingulate cortex

**DOI:** 10.1038/s41598-023-29140-8

**Published:** 2023-02-20

**Authors:** Ryo Kawabata, Shuji Shimoyama, Shinya Ueno, Ikuko Yao, Akiko Arata, Kohei Koga

**Affiliations:** 1 Department of Biomedical Chemistry major, Graduate School of Science and Technology, Kwansei Gakuin University, Sanda, Hyogo Japan; 2 Department of Neurophysiology, Hyogo Medical University, Nishinomiya, Hyogo Japan; 3 Department of Neurophysiology, Hirosaki University Graduate School of Medicine, Hirosaki, Aomori Japan; 4 Department of Physiology, Hyogo Medical University, Nishinomiya, Hyogo Japan

**Keywords:** Neuroscience, Physiology

## Abstract

Transient receptor potential ankyrin 1 (TRPA1) is a member of the TRP channel family and is expressed in peripheral and central nervous systems. In the periphery, TRPA1 senses cold and pain. However, the functions of TRPA1 in the CNS are unclear. Here, we examined the roles of TRPA1 on neural activity and synaptic transmission in layer II/III pyramidal neurons from mice anterior cingulate cortex (ACC) by whole-cell patch-clamp recordings. The activation of Cinnamaldehyde (CA), which is TRPA1 agonist produced inward currents and these were blocked by the TRPA1 antagonists. Furthermore, activating TRPA1 changed the properties of action potentials such as the firing rate, rise time and decay time. In contrast, stimulating TRPA1 did not alter the spontaneous synaptic transmission. Finally, we examined the functional role of TRPA1 on neurons in a hypoxic environment. We induced an acute hypoxia by substituting nitrogen (N_2_) gas for oxygen (O_2_) in the external solution. N_2_ produced biphasic effects that consisting of inward currents in the early phase and outward currents in the late phase. Importantly, blocking TRPA1 reduced inward currents, but not outward currents. In contrast, a K_ATP_ channel blocker completely inhibited outward currents. These results suggest that TRPA1 acts on postsynaptic neurons in the ACC as an acute O_2_ sensor.

## Introduction

Transient receptor potential (TRP) ion channels are cationic non-selective and ligand-gated. These channels have emerged as an evolutionarily conserved family and function as molecular detectors of physiological stimuli for various environments^1^. Among TRP channels, transient receptor potential ankyrin 1 (TRPA1) channels broadly distribute in the peripheral and the central nervous systems (CNS) including dorsal root ganglion, trigeminal ganglion and the brain^1,2^. TRPA1 expression is found in neurons and astrocytes^1,3,4^. TRPA1 is well-known as a channel for responding to cold temperature and pain in the peripheral nervous system^1^. TRPA1 is expressed in peripheral sensory pathways, where they act as mechanosensors to transducer of cold and nociceptive stimuli, and play important roles in the generation and pathological pain^1,2^. Remarkably, TRPA1 can act as a biosensor for oxygen (O_2_) concentration in a complex manner^5^.

The regulation of energy metabolism is indispensable to the central nervous system where energy consumption is a highly dynamic. In the brain, increased neuronal activity drives enhanced energy consumption^6^, with requires increased molecular O_2_ for metabolism. Under normal conditions, the brain requires ~ 20% of total body O_2_ consumed in adult human^7^. Previous studies demonstrate that astrocytes in the cortex are important for O_2_ sensing^8^. Notably, neurons consume approximately 75–80% of energy produced in the brain. Increased neural activity drives increase energy consumption in the brain. O_2_ is required for the generation of adenosine triphosphate (ATP) which is a major source of energy in aerobic organisms. The O_2_ and ATP demand for neural activity including maintenance of resting membrane potentials (RMPs), generation of action potentials (APs) and synaptic inputs^9^. The interactions of synapses and neurons are the foundation of neural circuits and ultimately give rise to higher order brain functions^10^.

The anterior cingulate cortex (ACC) is a part of the cerebral cortex and plays an important role in various higher brain functions including emotions, cognition, and pain^11–13^. For example, anxiety like behaviors (e.g. elevated plus maze) in mice can induce long-term synaptic plasticity in the ACC^11,14^. This plasticity consists of a presynaptic form of long-term potentiation in layer II/III pyramidal neurons of the ACC^12,15^. Additionally, chronic inflammation and peripheral nerve injury enhance the release of glutamate in the ACC^16–18^. By contrast, inflammatory pain reduces the release of GABA in the ACC^19^. The activation of the pyramidal neurons within the ACC by optogenetic manipulations elicit mechanical hypersensitivity in control as well as in animals with chronic pain^20^. Interestingly, the ACC also responds to the experience of breathlessness. Indeed, a human imaging study has demonstrated that the condition of acute hunger for air is capable to activate the ACC^21^. However, the role of TRPA1 on neural activity and synaptic transmission in the ACC is still unclear. Furthermore, the functional role of TRPA1 have not been investigated in the ACC.

In this study, we examine the role of TRPA1 on neural activity including APs and RMPs in layer II/III pyramidal neurons from the ACC by whole-cell patch-clamp recordings under acute brain slice preparations in vitro. We next determined whether or not TRPA1 could contribute to both glutamatergic and inhibitory synaptic transmission. Furthermore, we studied the functional role of TPRA1 following acute hypoxia in response to bath application of N_2_ gas.

## Results

### Activation of TRPA1 produced inward currents in the ACC

**Figure 1:**
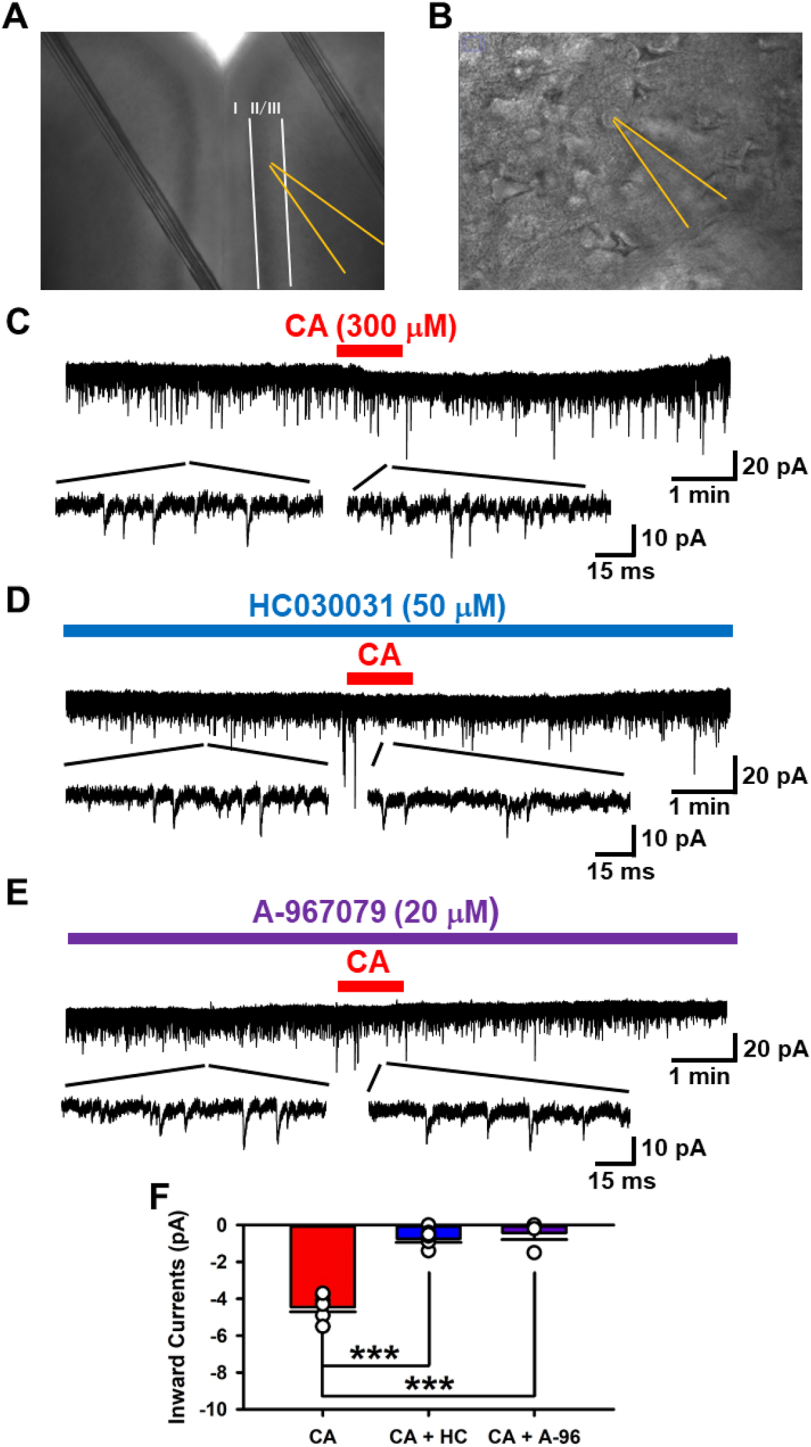
TRPA1 drives inward currents in the ACC. (**A**) Image of the ACC region from which whole-cell patch-clamp was performed, observed under the Infrared microscope (5x). (**B**)Image of layer II/III pyramidal neurons of the ACC under the Infrared microscope (40 ×). (**C**) Sample trace of sEPSCs: Inward currents occurred for 1 min perfusion of a TRPA1 agonist, cinnamaldehyde (CA: 300 μM). (**D**) Sample trace of sEPSCs in the presence of a TRPA1 antagonist, HC030031 (HC: 50 μM). (**E**) Sample trace of sEPSCs in the presence of a TRPA1 antagonist, A-96079 (A-96: 20 μM). (**F**) Averaged inward currents of sEPSCs in the presence of CA and CA + HC. In the presence of HC, inward currents were decreased (CA: − 4.6 ± 0.3 pA, n = 7 neurons/7 mice; CA + HC: − 0.7 ± 0.2 pA, n = 7 neurons/7 mice; CA + A-96:). ***P = 0.001 compared with CA (one-way ANOVA, F_2,15_ = 80.887). Data are presented as mean ± SEM.

First, using whole-cell patch-clamp recordings from brain coronal slices, we examined if pharmacological activations of TRPA1 by Cinnamaldehyde (CA) could change the baseline currents in pyramidal neurons of layer II/III in the ACC of adult mice (Fig. 1A,B). Holding membrane potentials at − 70 mV, we recorded sEPSCs. Three minutes after the stable recording, CA (300 μM) was applied for 1 min in the bath solution^22^. The application of CA led to inward currents (CA: − 4.5 ± 0.3 pA, n = 7 neurons/7 mice, Fig. 1C). Subsequently, the CA-induced inward currents were inhibited in the presence of a TRPA1 antagonist, HC030031 (HC, 50 μM) (CA + HC; − 0.74 ± 0.2 pA, n = 7 neurons/7 mice, Fig. 1D). Consistently, the selective TRPA1 antagonist, A-96079 (A-96, 20 μM) was found to block CA-induced inward currents in the ACC (CA + A-96: − 0.45 ± 0.4 pA, n = 4 neurons/3 mice, Fig. 1E). Therefore, the acute application of CA-induced inward currents was mediated by TRPA1 (one-way ANOVA, F_2,15_ = 80.887, *P* = 0.001, Fig. 1F). These results suggest that the acute generation of the inward currents were mediated by activations of TRPA1.

### Activation of TRPA1 did not affect synaptic transmissions

**Figure 2:**
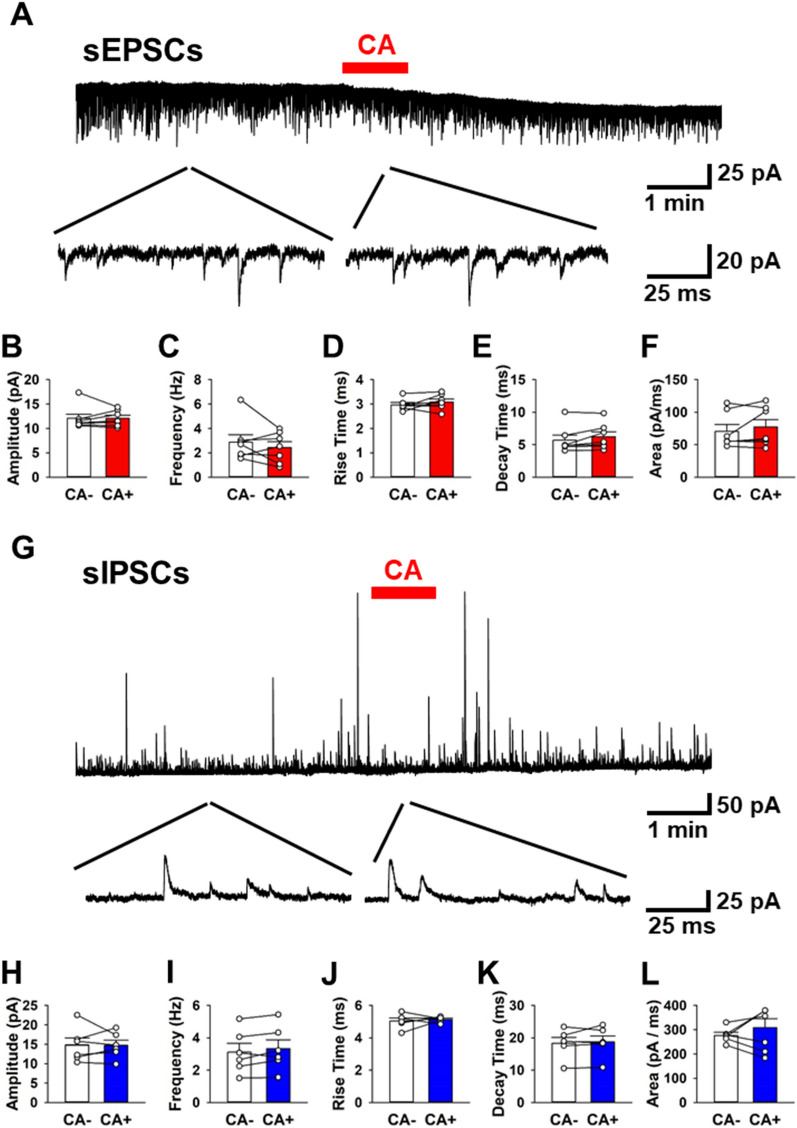
TRPA1 is unrelated to both excitatory and inhibitory synaptic transmission. (**A**) Sample trace of sEPSCs. CA: cinnamaldehyde (300 μM). (**B**–**F**) Averaged amplitude (**B**), frequency (**C**), rise time (**D**), decay time (**E**) and area (**F**) of sEPSCs before and after CA (B; CA − : 12.0 ± 0.9 pA; CA + : 12.0 ± 0.6 pA, t (6) =  − 0.0108, *P* = 0.496, C; CA − : 2.9 ± 0.6 Hz; CA + : 2.4 ± 0.5 Hz, t (6) = 0.578, *P* = 0.289, D; CA − : 3.0 ± 0.1 ms; CA + : 3.1 ± 0.1 ms, t (6) =  − 0.75, *P* = 0.234, E; CA − : 5.7 ± 0.8 ms; CA + : 6.2 ± 0.8 ms, t (6) =  − 0.474, P = 0.322, F; CA − : 70.5 ± 10.2 pA/ms; CA + : 77.2 ± 11.3 pA/ms, t (6) =  − 0.439, *P* = 0.334, n = 7 neurons/7 mice). (**G**) Sample trace of sIPSCs. (**H**–**L**) Averaged amplitude (**H**), frequency (**I**), rise time (**J**), decay time (**K**) and area (**L**) of sIPSCs before and after CA (H; CA − : 14.8 ± 1.8 pA; CA + : 14.7 ± 1.3 pA, t (5) = 0.0478, *P* = 0.481, I; CA − : 3.1 ± 0.5 Hz; CA + : 3.3 ± 0.6 Hz, t (5) =  − 0.276, *P* = 0.394, J; CA − : 5.0 ± 0.2 ms; CA + : 5.1 ± 0.1 ms, t (5) =  − 0.55, P = 0.297, K; CA − : 18.3 ± 1.8 ms; CA + : 18.7 ± 1.9 ms, t (5) =  − 0.139, *P* = 0.446, L; CA − : 276.5 ± 12.5 pA/ms; CA + : 308.9 ± 36.4 pA/ms, t (5) =  − 0.411, *P* = 0.345, n = 6 neurons/6 mice). All tests for Figure were performed by paired *t* test. Data are presented as mean ± SEM.

Next, we tested if the stimulation of TRPA1 by CA could alter synaptic transmissions on both spontaneous excitatory and inhibitory synapses in the ACC (Fig. 2A). Since sEPSCs were completely abolished by the AMPA/GluK antagonist (CNQX), sEPSCs must be mediated via AMPA/GluK receptors^23^. We analyzed the electrophysiological property of sEPSCs before and after application of CA (n = 7 neurons/7 mice). The average amplitudes of sEPSCs were not changed between CA − and CA + groups (Fig. 2B). The average frequency of sEPSCs was also unaffected between the two groups (Fig. 2C). In addition, we analyzed the electrophysiological property of sEPSCs including the rise time, decay time and area of the current curve. There was no difference in any of the parameters between the two groups (Fig. 2D,E,F).

We also analyzed if CA could alter the electrophysiological property of sIPSCs. Since the sIPSCs were completely abolished by the GABA_A_ receptor antagonist (picrotoxin), sIPSCs were mediated through GABA_A_ receptors^23^. We analyzed the property of sIPSCs before and after CA (n = 6 neurons/6 mice, Fig. 2G). The average amplitude of sIPSCs remained unchanged between the groups (Fig. 2H). The average frequency of sIPSCs was not altered between the groups (Fig. 2H). Furthermore, we analyzed rise time, decay time, and area of sIPSCs. Rise time, decay time and area were not altered (Fig. 2H,I,J,K,L). These results indicate that CA-induced activation of TRPA1 may not be mediated through neither excitatory nor inhibitory synaptic transmission in the layer II/III pyramidal neurons of the ACC.

### Effect of TRPA1 on the production of APs and RMPs

**Figure 3:**
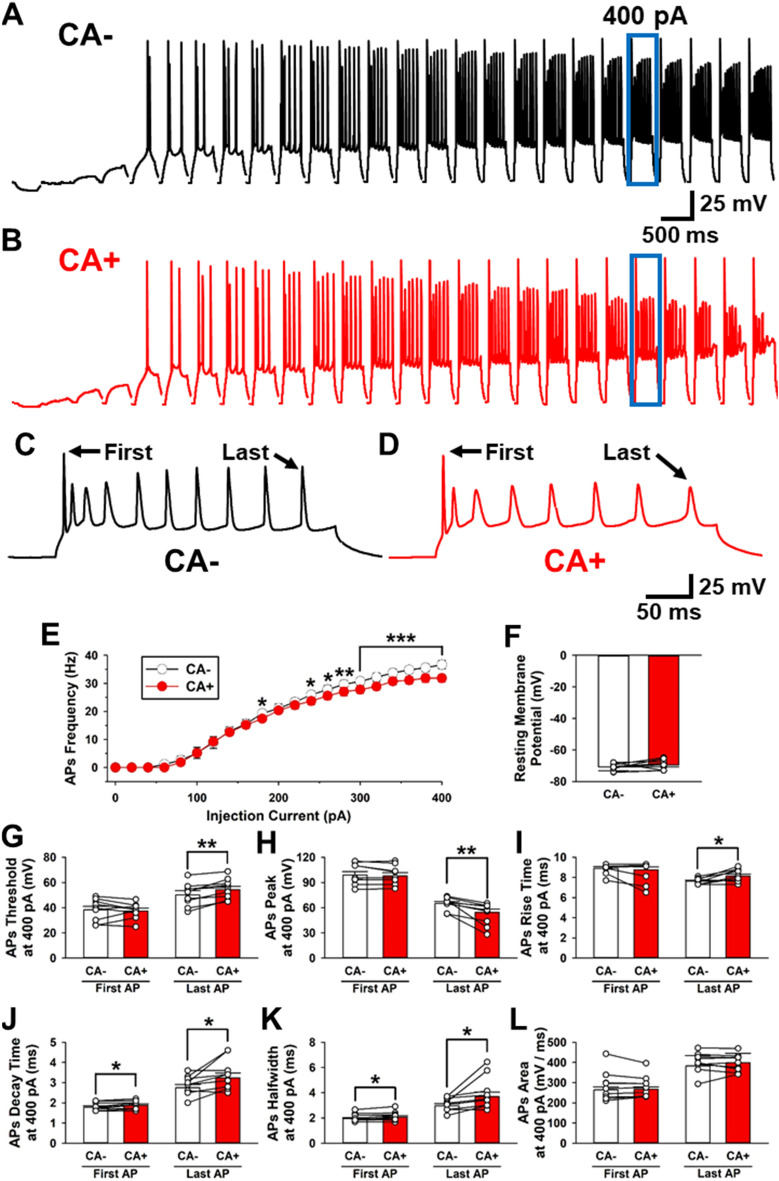
TRPA1 affects the shape and frequency of APs. (**A–B**) Sample trace of APs in CA − (**A**) and CA + (**B**) by current injections (300 ms duration from 0 to 500 pA, every + 20 pA step). (**C**–**D**) Sample trace of APs that were stimulated by 400 pA current in CA − (**C**) and CA + (**D**). The APs generated at the beginning and end of the current step these were analyzed. (**E**) Averaged frequency of APs. Activation of TRPA1 reduced the frequency of APs with high currents injection (n = 9 neurons/9 mice, two-way-repeated measure ANOVA, F_1,160_ = 10.252, *P* = 0.013). (**F**) Averaged RMPs before and after CA. RMP did not change CA − and CA + (CA − : − 70.8 ± 0.8 mV; CA + : − 69.5 ± 1.2 mV, paired *t* test, t (8) =  − 1.288, *P* = 0.234). (**G**) Averaged threshold of first and last APs. Threshold of first APs had no change (CA − : − 38.2 ± 2.9 mV; CA + : − 37.4 ± 2.1 mV, paired *t* test, t (8) = 0.471, *P* = 0.651), but threshold of last APs was raised (CA − : − 50.4 ± 0.2 mV; CA + : − 55.2 ± 2.6 mV, paired *t* test, t (8) =  − 4.368, *P* = 0.002). (**H**) Averaged peak of first and last APs. Perfusion of CA did not affect the first APs (CA − : 98.9 ± 4.2 mV; CA + : 97.9 ± 3.9 mV, paired *t* test, t (8) = 1.425, *P* = 0.192), but reduced to the last APs (CA − : 65.1 ± 2.3 mV; CA + : 54.5 ± 3.5 mV, paired t-test, t (8) = 3.857, *P* = 0.005). (**I**) Averaged rise time of first and last APs. Rise time of first APs did not change (CA − : 8.9 ± 0.2 ms; CA + : 8.8 ± 0.3 ms, paired *t* test, t (8) = 1.418, *P* = 0.194), but rise time of last APs was delayed (CA − : 7.6 ± 0.1 ms; CA + : 8.1 ± 0.2 ms, paired *t* test, t (8) =  − 3.303, P = 0.011). (**J**) Averaged decay time of first and last APs. Decay time of first APs was unchanged (CA − : 1.8 ± 0.1 ms; CA + : 1.9 ± 0.1 ms, paired *t* test, t (8) =  − 2.8, *P* = 0.023), but decay time of last APs was delayed (CA − : 2.8 ± 0.2 ms; CA + : 3.2 ± 0.2 ms, paired t-test, t (8) =  − 2.689, *P* = 0.028). (**K**) Averaged area of first and last APs. Both area of first APs and last APs were unchanged. (First APs; CA − : 263.8 ± 15.0 mV/ms; CA + : 266.3 ± 11.6 mV/ms, paired t-test, t (8) = 0.404, *P* = 0.697, Last APs; CA − : 191.7 ± 8.5 mV/ms; CA + : 199.1 ± 7.8 mV/ms, paired *t* test, t (8) = 0.251, **P** = 0.808). (**L**) Averaged halfwidth of first and last APs. There was no change in halfwidth of first APs (CA − : 1.95 ± 0.07 ms; CA + : 2.02 ± 0.08 ms, paired *t* test, t (8) =  − 3.035, P = 0.016), but last APs widened (CA − : 3.0 ± 0.2 ms; CA + : 3.7 ± 0.3 ms, paired *t* test, t (8) =  − 2.511, *P* = 0.036).

In order to analyze the roles of TRPA1 on neural activity, we examined if stimulating TRPA1 by CA could change the production of APs and RMPs (Fig. 3). The APs were generated by current injections (300 ms duration, from 0 to 500 pA, + 20 pA steps, Fig. 3A). The frequency and shape of APs as well as RMPs were compared between the CA − and CA + groups (n = 9 neurons/9 mice, Fig. 3). The activation of TRPA1 changed the generation and shapes of APs with injection of high current intensities of 400 pA (Fig. 3A,B,C,D). The activation of TRPA1 by CA decreased the frequency of APs (Fig. 3E). Conversely, there was no significant difference observed when comparing the stimulated (CA +) TRPA1 RMP to the non-stimulated (CA −) TRPA1 RMP (Fig. 3F).

Next, we analyzed whether CA stimulation could change the shapes of APs. We examined the first and last APs generated with high current injections (400 pA). The average threshold of the last APs was raised after CA stimulation, when compared to the non-stimulated TRPA1 (Fig. 3G). Furthermore, the average peak amplitude of the last AP was suppressed following CA stimulation (Last AP: CA − : 65.1 ± 2.3 mV; CA + : 54.5 ± 3.5 mV, paired *t* test, t (8) = 3.857, *P* = 0.005, Fig. 3H). The average rise time of last AP was delayed after CA stimulation (Last AP: CA − : 7.6 ± 0.1 ms; CA + : 8.1 ± 0.2 ms, paired *t* test, t (8) =  − 3.303, *P* = 0.011, Fig. 3I). Additionally, the average decay time of the first and last AP was delayed (First AP: CA − : 1.8 ± 0.1 ms; CA + : 1.9 ± 0.1 ms, paired *t* test, t (8) =  − 2.8, *P* = 0.023; Last AP: CA − : 2.8 ± 0.2 ms; CA + : 3.2 ± 0.2 ms, paired *t* test, t (8) =  − 2.689, *P* = 0.028, Fig. 3J). However, there was no difference in the average area of APs when comparing the stimulated CA + TRPA1 to the non-stimulated CA − TRPA1 (Fig. 3L). The average halfwidth of last APs was wider after CA stimulation (First AP: CA − : 1.95 ± 0.07 ms; CA + : 2.02 ± 0.08 ms, paired *t* test, t (8) =  − 3.035, *P* = 0.016; Last AP: CA − : 2.8 ± 0.2 ms; CA − : 3.0 ± 0.2 ms; CA + : 3.7 ± 0.3 ms, paired *t* test, t (8) =  − 2.511, *P* = 0.036, Fig. 3J). These results suggest that stimulation of TRPA1 influences the production of APs and their electrophysiological properties in the ACC.

### Roles of TRPA1 and K_ATP_ channels on N_2_-induced acute hypoxia

**Figure 4:**
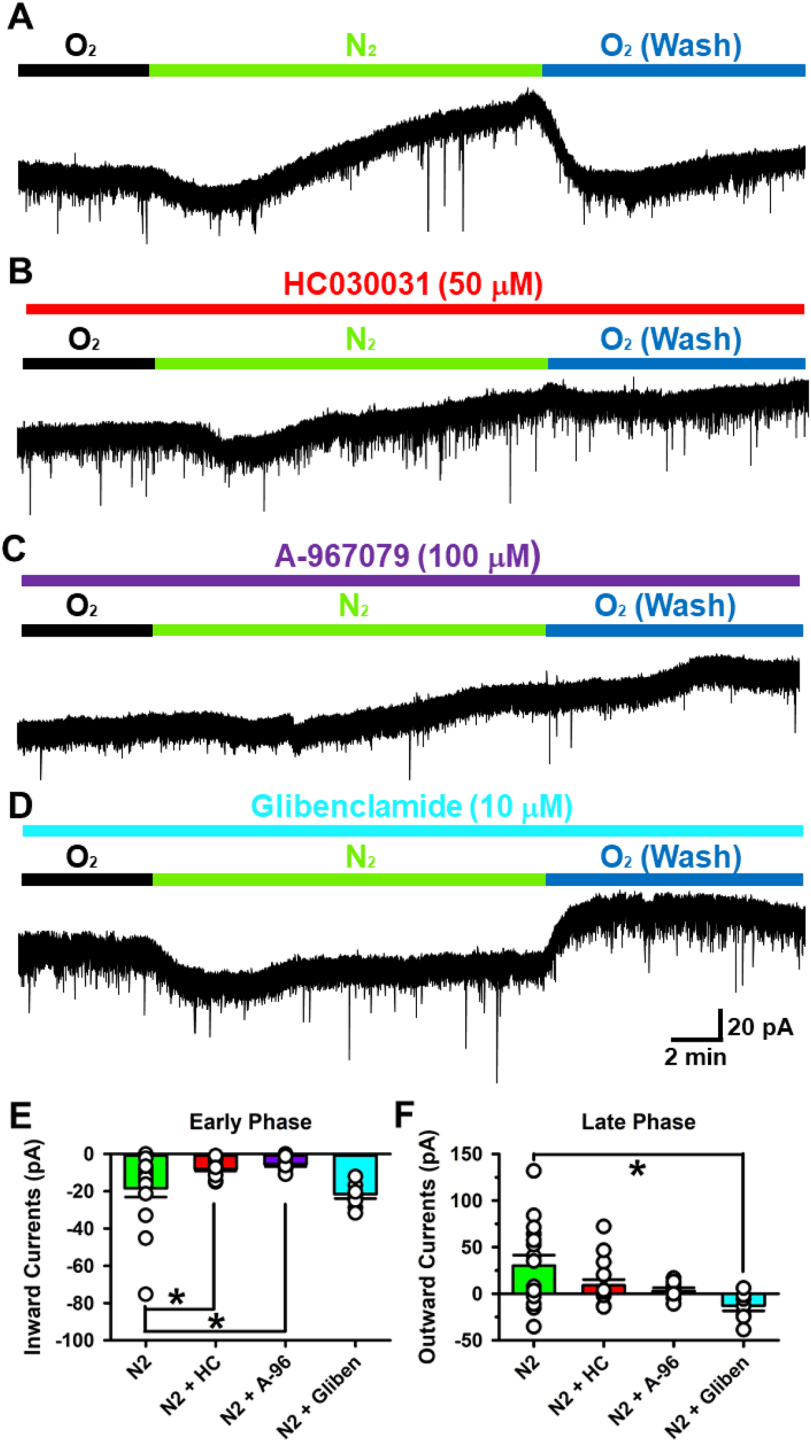
TRPA1 senses oxygen in ACC. (**A**) Sample trace of sEPSCs by N_2_ induced hypoxia. (**B**) Sample trace of sEPSCs in the presence of HC030031 in N_2_-hypoxia. Inward currents were decreased in the early phase. (**C**) Sample trace of sEPSCs in the presence of A-967076 in N_2_-hypoxia. Inward currents were also decreased in the early phase. (**D**) Sample trace of sEPSCs in the presence of a K_ATP_ channel blocker, Glibenclamide (10 μM) in hypoxia. (**E**) Averaged inward current. Inward current decreased only in the presence of HC030031 (N_2_: − 18.4 ± 4.7 pA, 16 neurons/15 mice; N_2_ + HC: − 7.9 ± 1.2 pA, 15 neurons/12 mice; N_2_ + A-96: − 5.4 ±3.0 pA, n = 8 neurons/6 mice; N_2_ + Gliben: − 21.6 ± 2.3 pA, 9 neurons/8 mice, one-way ANOVA, F_3,44_ = 5.416, *P* = 0.003). (**F**) Averaged outward current. Outward current completely decreased only in the presence of Glibenclamide (N_2_: 30.3 ± 11.1 pA, 16 neurons/15 mice; N_2_ + HC: 8.9 ± 6.3 pA, 15 neurons/12 mice; N_2_ + A-96: 3.0 ± 3.4 pA, 8 neurons/6 mice; N_2_ + Gliben: − 13.0 ± 5.5 pA, 9 neurons/8 mice, one-way ANOVA, F_3,44_ = 4.703, *P* = 0.006).

Finally, we studied the functional role of TRPA1 in the ACC (Fig. 4). Hypoxia is a condition in which O_2_ is not available in sufficient quantity to maintain adequate homeostasis at the tissue level^24^. To produce acute hypoxia, we replaced 95% O_2_ gas with 95% N_2_ in the bath solution^25^. 95% N_2_ gas produced biphasic effects in the ACC (Fig. 4A). In the early phase, N_2_ produced inward currents (− 8.4 ± 4.7 pA, 16 neurons/15 mice). In the late phase, N_2_ caused outward currents (30.3 ± 11.1 pA, Fig. 4A). In the presence of a TRPA1 antagonist (HC), we replaced O_2_ with N_2_ and found that HC030031 inhibited inward currents in the early phase (− 7.9 ± 1.2 pA, one-way ANOVA, F_3,44_ = 5.416, *P* = 0.0037 compared with N_2_, n = 15 neurons/12 mice), but had no effect on the late phase response (Fig. 4B, F). Blocking TRPA1 by A-967079 (100 μM) also reduced the N_2_-induced inward currents in the early phase (− 5.4 ± 3.0 pA, one-way ANOVA, F_3,44_ = 5.416, *P* = 0.0039 compared with N_2_, n = 8 neurons/6 mice, Fig. 4C, E). Furthermore, we explored the mechanism of outward currents in the late phase (Fig. 4D). Since O_2_ helps the generation of ATP, it is possible that K_ATP_ channels may be related to the outward currents^25^. A K_ATP_ channel blocker, Glibenclamide (Gliben, 10 μM) completely blocked outward current in the late phase (− 13.0 ± 5.5 pA, one-way ANOVA, F_3,44_ = 4.703, *P* = 0.006 compared with N_2_, n = 9 neurons/8 mice, Fig. 4D,F).

**Figure 5:**
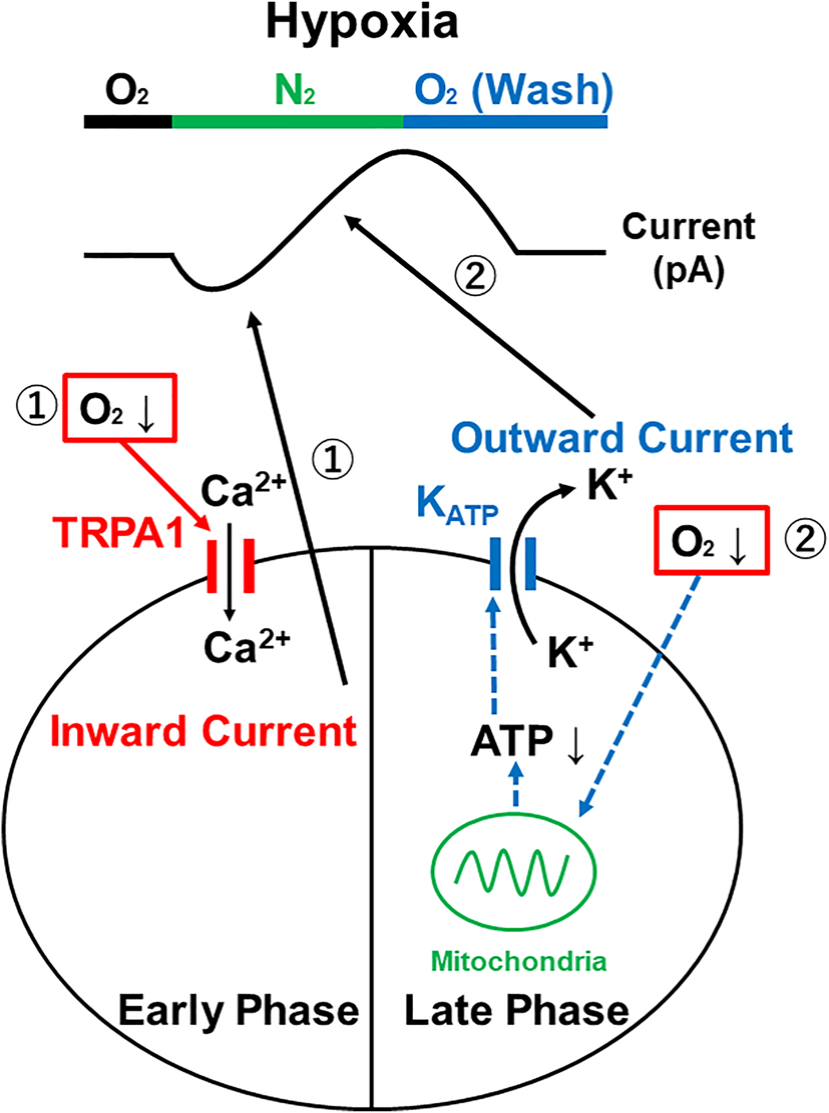
A possible mechanism on acute hypoxia in the ACC. Replacement of O_2_ to N_2_ induced acute hypoxia in the ACC. The acute hypoxia produced the biphasic effects of inward currents in the early phase (1) and outward currents in the late phase (2). (1) In the early phase, TRPA1 senses extracellular O_2_ depletion and TRPA1 opens. Opening TRPA1 allows cations such as Ca^2+^ to flow into the cell generating inward currents. (2) In the late phase, decreased O_2_ results in a decreased production of intracellular ATP. The decreasing ATP concentration causes an opening of K_ATP_ channels and efflux of K^+^ to the extracellular environment. The opening of K_ATP_ channels generates the outward currents observed.

These results suggest that TRPA1 plays a role in N_2_-induced inward response in the early phase and that the K_ATP_ channel is critical for the outward currents in the late phase under conditions of acute hypoxia (Fig. 5).

## Discussion

We studied the roles of TRPA1 on neural activity and synaptic transmission in the brain slice preparation by using whole-cell patch-clamp recording from adult mice ACC neurons. TRPA1 affected neural activity, but not synaptic transmission. Furthermore, we examined the functional role of TRPA1 on N_2_ gas-induced hypoxia. Importantly, neural TRPA1 is involved in the early phase of N_2_ induced hypoxia in the cortex. A previous study has reported that cortical astrocytes also play a role in hypoxia sensing^8^. Hypoxia is also sensed in the parafacial respiratory group and retrotrapezoid nucleus, composing the medullary respiratory center. TRPA1 in these regions were predominantly found in astrocytes and critically involved in O_2_ sensing, but not in choline acetyltransferase positive neurons^26^. In light of the current investigation, it is likely that sensing O_2_ may be cell type and region specific^8,26^.

It has been reported that TRPA1 distributes across vessels, astrocytes and neurons^3,27^. Anatomical studies show that TRPA1 is expressed in peripheral and the CNS, including the spinal cord, hippocampus, amygdala and cortical regions^1,3,28^. In the CNS, TRPA1 plays various roles in synaptic transmissions and/or neural activity depending on the specific brain region. For example, in the spinal cord and the supraoptic nucleus, activations of TRPA1 facilitate the release of neurotransmitter^28,29^. Our work showed that activation of TRPA1 did not alter synaptic transmissions in the ACC. Stimulating TRPA1 did not change the spontaneous synaptic transmission for both excitatory and inhibitory inputs. Since the frequency of sEPSCs and sIPSCs were unchanged, TRPA1 may not affect the release of glutamate nor GABA in the cortex. In contrast, activations of TRPA1 by CA altered the generation as well as electrophysiological properties of APs.

Therefore, within the ACC, TRPA1 is likely to have a postsynaptic role. Consistently, confocal imaging shows TRPA1 expression in the soma and dendrites of neurons across all cortical layers^4^. Furthermore, it is reported that many TRPA1-expressing neurons co-express TRPV1^30^. Importantly, TRPV1 and TRPA1 may also be co-expressed in neurons of the ACC^31^. However, we cannot exclude the possibility that local activation of TRPA1 expressing astrocytes or microglia enhance neuronal excitability through the increase of intracellular calcium in astrocytes as has been demonstrated for other brain regions^3,32^. Further experiments are needed to identify the roles of TRPA1 in neurons and/or astrocytes.

We induced APs in ACC neurons by injecting currents from 0 to 500 pA. The current injections produced APs, and applications of CA reduced APs at high current injections. Furthermore, the property of APs including rise time, decay time, half width and the peak were altered by CA at high currents injections (400 pA). In particular, CA reduced the peak of APs in the cortex. However, activation of TRPA1 by CA did not alter the RMPs. These results are comparable with previous studies in the primary somatosensory cortex or frog sciatic nerve^22,33^.

How activations of TRPA1 alter the electrophysiological properties of APs is not yet fully understood. Since APs are a product of the ion flux driven by voltage-gated sodium and potassium currents, activation of TRPA1 may influence the action of channels controlling these conductances. For example, the TRP channels TRPV1 and TRPM8 are in the same family as TRPA1. Notably, activations of TRPV1 by capsaicin or TRPM8 by menthol lead to a suppression of voltage-gated sodium channels, resulting in an inhibitory effect on APs^34,35^. Thus, similar effects may happen by stimulating TRPA1 on APs via voltage-gated sodium channels and affect the rise time and the peak of APs.

Replacement of O_2_ with N_2_ gas in bath solution produced hypoxia^25^. In our study, N_2_ produced biphasic effects in the ACC neurons. The biphasic response consisted of inward currents in the early phase and outward currents in the late phase. These results are similar to that of a previous study of the locus coeruleus^25^. Importantly, a TRPA1 antagonist reduced inward currents in the early phase, but not outward currents in the late phase. On the other hand, the outward currents in the late phase were inhibited by ATP-sensitive potassium channels (K_ATP_) channel blocker, Glibenclamide. Since blocking TRPA1 did not alter N_2_-induced outward currents, the inward and outward currents may have different mechanisms. Acute removal of O_2_ may change membrane currents through several pathways. One possible explanation is that the inward currents produced in the early phase were due to TRPA1 sensing the reduction of O_2_, resulting in an immediate opening of the channel. The late phase was the result of long lasting-depletion of O_2_. This depletion may reduce the generation of ATP in mitochondria since O_2_ is directly involved in the production of ATP in mitochondria^36^. The amount of ATP can control the gating of K_ATP_ channel^25^. Thus, lower levels of ATP, due to depletion of O_2,_ may lead to the opening of K_ATP_ channels.

It has reported that the ACC responses associated with consciousness of breathlessness in human. A positron emission tomography study shows that the condition of acute hunger for air after inhalation of carbon dioxide activate the ACC region^21^. An animal study has also demonstrated that TRPA1 is involved in hypoxia-induced behaviors in mice^37^. For instance, *TRPA1* knockout (KO) mice have attenuated avoidance behaviors in response to a low O_2_ environment and ventilator responses to mild hypoxia. In the peripheral nervous system, TRPA1 is activated by hypoxia^38^, The deficiencies in tissue O_2_ cause comorbidity in higher brain functions, aging and disease. For example, reduction of O_2_ by ischemic stroke affect higher brain functions such as learning and memory^24^. The metabolism of O_2_ is also related to diseases such as stroke and ischemia^24^. Ischemic stroke happens when the blood supply to the brain is obstructed, preventing brain tissue from getting O_2_ and nutrients. These diseases directly affect higher brain functions, such as cognition and speech, where the ACC is involved. Therefore, it is possible that the functions of TRPA1 as an O_2_ sensor in the ACC may also play a role in these systems.

## Conclusion

The activation of TRPA1 produced inward currents and changed active membrane property in the mice ACC. Acute hypoxia by substituting N_2_ gas for O_2_ in the external solution caused biphasic effects that consisting of inward currents in the early phase and outward currents in the late phase. TRPA1 and K_ATP_ channel play roles in inward currents and outward currents, respectively. These results suggest that TRPA1 acts on postsynaptic neurons in the ACC as an acute O_2_ sensor.

## Materials and methods

### Study approval

The present study protocol was approved by the Ethics Committee of Hyogo Medical University (Approval No. 22–002). Processes of all animal experiments were confirmed by Hyogo Medical University Committee on Animal Research and were conducted in accordance with ARRIVE guidelines.

### Animal

Male adult C57BL/6 J mice (8–15 weeks old) were used in the experiment. Mice were housed at 23 ± 2 °C with a 12/12-h dark cycle (light on at 07:00 h) and were supplied access to general feed and water freely. Every effort was made to relieve animals of suffering and reduce the number of animals used as much as possible.

### Whole-cell patch-clamp recording in pyramidal cells of ACCs from coronal slice

Coronal brain slices (300 μm) were prepared including the ACC region by a vibratome (7000smz-2, Campden, Loughborough, Leics, England)^12^. Brain slices were put in a chamber for storing slices filled with oxygenated (95% O_2_ and 5% CO_2_) artificial cerebrospinal fluid (ACSF) containing (in mM) 124 NaCl, 2.5 KCl, 2 CaCl_2_, 1 MgSO_4_, 25 NaHCO_3_, 1 NaH_2_PO_4_, and 10 glucose at room temperature for about 1 h. All experiments were performed in a recording chamber on the stage of a BX51WI microscope (Olympus, Center Valley, PA, USA) with infrared differential interference contrast optics for neuron visualization. Whole-cell patch clamp recordings were performed from layer II/III pyramidal cells in the ACC with an amplifier (IPA, Sutter Instrument, Novato, CA, USA) at room temperature. In the voltage-clamp and current-clamp modes, tip microelectrodes were filled with internal liquid constituted of (in mM) 120 K-gluconate, 5 NaCl, 1 MgCl_2_, 0.5 EGTA, 2 Mg-ATP, 0.1 Na_3_GTP, and 10 HEPES; pH 7.2, 280–300 mosmol were used for spontaneous excitatory postsynaptic currents (sEPSCs), resting membrane potentials (RMPs) and action potentials (APs). For recording sEPSCs, the holding membrane was kept at − 70 mV in voltage-clamp mode with a GABA_A_ receptors blocker, picrotoxin (100 μM) in ACSF continuously. APs were recorded by adjusting the membrane potentials about − 70 mV by changing the holding currents in the current-clamp mode. When spontaneous inhibitory postsynaptic currents (sIPSCs) were recorded, tip microelectrodes were filled with internal liquid constituted of (in mM) 120 Cs-gluconate, 5 NaCl, 1 MgCl_2_, 0.5 EGTA, 2 Mg-ATP, 0.1 Na_3_GTP, and 10 HEPES; pH 7.2, 280–300 mosmol. To record sIPSCs, holding membrane potential was kept at 0 mV in voltage-clamp mode. The APs, sEPSCs and sIPSCs were analyzed by Mini Analysis Software, and membrane potentials were analyzed by Clampfit 10.7. The rise and decay time of APs, sEPSCs and sIPSCs were monitored between 10 and 90% of their peak and amplitude.

### Drugs

Cinnamaldehyde (CA), HC030031 (HC), A-967079 (A-96), picrotoxin, CNQX and Glibenclamide (Gliben) were purchased from Fujifilm. CA, picrotxin was dissolved in alcohol, and HC, CNQX and Gliben were dissolved in DMSO as a stock solution.

### Statistics

Graphs were created with SigmaPlot12.5. Statistical analyses were done using the statistical function in SigmaPlot12.5. For comparison before and after reagent administration in the same recorded neurons, paired-*t* test was performed. Unpaired-*t*-test was used for comparison between different recorded neurons. Data with more than two groups were compared using one-way or two-way analysis of variance (ANOVA), followed by the Bonferroni post hoc test. All data were represented average ± SEM. Difference was considered significant at *P* < 0.05.

## Data Availability

The datasets used and/or analyzed during the current study available from the corresponding author, K.K., on reasonable request.

## Abbreviations

A-96: A-967079
ACC: Anterior cingulate cortex
ACSF: Artificial cerebrospinal fluid
APs: Action potentials
ATP: Adenosine triphosphate
CA: Cinnamaldehyde
CNS: Central nervous system
Gliben: Glibenclamide
HC: HC030031
Nitrogen: N_2_
Oxygen: O_2_
RMP: Resting membrane potential
sEPSCs: Spontaneous excitatory postsynaptic currents
sIPSCs: Spontaneous inhibitory postsynaptic currents
TRP: Transient receptor potential
TRPA1: Transient receptor potential ankyrin 1
